# Future Scenarios of the Data-Driven Healthcare Economy in South Korea

**DOI:** 10.3390/healthcare10050772

**Published:** 2022-04-21

**Authors:** Ji-Young Choi, Hee-Jo Lee, Myoung-Jin Lee

**Affiliations:** 1 BK21FOUR R&E Center for Learning Health Systems, Korea University, Seoul 02841, Korea; pettie1@korea.ac.kr; 2 Department of Sociology, Korea University, Seoul 02841, Korea; leehj913@korea.ac.kr

**Keywords:** healthcare, digital, data, general morphological analysis, scenario technique

## Abstract

Although data-based healthcare innovation has been spotlighted in South Korea in recent years, previous studies have made little effort to systematically predict various possible future outcomes in the data-driven healthcare economy. This study investigated possible future such scenarios in South Korea by conducting a general morphological analysis (GMA). Seven key factors were identified that will drive the data-driven healthcare economy: the acceptability of data utilization, the level of data literacy, the status of healthcare data regulation, the healthcare data system, medical costs, the convergence of ICT and biotechnology, and the utilization of data in medical services. The main findings are as follows: Four possible scenarios for the data-driven healthcare economy in South Korea were identified. The first scenario suggested mostly optimistic prospects and close associations between factorial values on the various spectra. The second scenario was similar to the first one, except for medical costs. However, the third scenario contrasted with the first, as it entailed relatively pessimistic factorial values. Finally, most of the elements of the current healthcare status quo were maintained in the fourth scenario. This study makes not only an academic contribution, but also has policy implications based on the four scenarios.

## 1. Introduction

The development of digital technology has created new services in various fields. The concept and reality of the ‘digital’, represented by the computer, the Internet, and the mobile phone, have become ubiquitous. However, new technologies have enabled the digital to become even more ever-present. These technologies include a range of portable devices, wireless local area networks inside buildings, and Code Division Multiple Access (CDMA) technologies outside buildings [1]. Beyond the simple utilization of digital technology, our everyday lives are digitalized and connected in the era of digital ubiquity. Individuals who are surrounded by densely connected technological infrastructure can gain online access without space–time constraints. Many scholars point out that this has led to the integration of diverse business models and encouraged a form of value creation whose purpose is to offer user-centered service [2,3,4].

Healthcare is emerging as a field in which new business opportunities are being created with the development and dissemination of digital technology. On the basis of this technology, individuals can self-monitor their health conditions more conveniently through their personal digital assistant (PDA) anytime and anywhere, without visiting a hospital. In fact, patients’ health can be managed without them even realizing it. Both PDAs and wireless internet function as a bridge between patients and doctors [1]. This shift contributes to the generation of new business visions and strategies in the healthcare sector.

In many countries, including the United States, Japan, and China, as well as in Europe, governments and private companies are already working on large-scale digital healthcare projects and attempting to enhance their competitiveness in the field of healthcare [5]. Moreover, since data are a key element of innovation in digital healthcare, these countries are paying a lot of attention to building data-based healthcare systems that could bring about effective changes not only in disease treatment, but also in prevention and care [6]. In other words, the significance of the data-driven healthcare economy is increasing. We define this economy as an emerging ecosystem that creates new value based on all types of data, and on data analysis related to healthcare. These countries are striving to devise policies at the national level in order to provide optimized medical services to individuals, by integrating healthcare data which are currently dispersed among medical institutions, administrative agencies, research institutes, and individuals [7].

Data-based healthcare system-building and digital healthcare industries are increasing in importance, particularly in East Asian countries. Their populations are aging at an unprecedented rate—especially in South Korea, where the older population is the fastest-growing in the world. This is placing an increasing burden on national finances [8]. In addition, the prevalence of chronic diseases such as high blood pressure and diabetes is rapidly growing in Korea, adding to medical expenses borne by the state [5]. Therefore, innovation must be driven by a data-driven healthcare system in order to reduce these expenses and to increase the quality and efficiency of healthcare. Although data-driven healthcare innovation is becoming more widespread and is growing in importance in South Korea, previous studies have not adequately analyzed nor focused on the potential circumstances of the data-driven healthcare economy in the future. Most studies have focused only on the technical aspect of digital healthcare or on its current state [9,10,11,12,13]. The future of the data-driven healthcare economy may not be to simply follow technological trends, as it may vary depending on how social, institutional, and technological factors related to healthcare data are combined with and connected to each other [5].

To fill these gaps in the field, this study investigated possible future scenarios of the data-based healthcare economy in South Korea. The research question for this study was ‘How will the data-driven healthcare economy unfold in South Korea in the future?’ More specifically, it analyzed various circumstances that could arise in Korea’s future healthcare data-driven economy with seven key factors in the area of healthcare data, namely the acceptability of data utilization, data literacy, healthcare data regulation, healthcare data system, medical costs, the convergence of ICT and biotechnology, and data utilization in medical services, and the degree of change in these factors. By examining these future scenarios, this study can contribute to our thinking about the appropriate measures that should be taken in this field, and the optimal decisions that should be made.

Several future scenarios of the data-driven healthcare economy were derived from this study’s general morphological analysis (GMA), which is a productive method of assessing various scenarios. GMA is based on processes of evaluation and on identifying internal consistency rather on than quantitative methods and the identification of causal relationships. When devising future scenarios or conducting policy analysis, political, economic, and social phenomena are difficult to assess through quantitative methodologies or on the basis of causality, as they entail unquantifiable elements and relationships which are usually uncertain and complex. Therefore, GMA was adopted as a solution to this methodological problem, as it was in fact established as a way of understanding and structurally conceptualizing multidimensional and unorganized problems [14].

## 2. Literature Review

### 2.1. Digital Healthcare Research

The social need for healthcare is expanding in many countries due to their aging populations, the improvement in living standards and consequently of expectations, and the increasing importance of the prevention and daily management of diseases. As ICT is combined with medical services to an ever-greater extent, the digital healthcare industry is in the spotlight as a business area that creates new value [2,3]. Accordingly, interest in the healthcare sector, especially digital healthcare, is increasing at the national level [4,7].

There are several definitions of digital healthcare, put forward by various scholars. Academic consensus on the definition and designation of what constitutes the integration of healthcare and ICT has yet to be reached, and so it has been disparately defined [15]. ‘Digital healthcare’ refers to the use of digital technologies in order to collect, share, and analyze information on health and wellbeing, which ultimately contributes to improving individual health and enhancing the quality of healthcare [16]. Another definition is as follows: ‘Digital healthcare refers to healthcare services in which data are collected, analyzed, and utilized through the convergence of technologies, including artificial intelligence (AI), big data, the Internet of Things, and cloud computing’ [17].

Although no complete consensus has been reached on the definition of digital healthcare, related studies have been conducted on various topics, as interest in the digital healthcare sector in Korean society has grown. Previous research conducted in South Korea has mainly identified trends in the digital healthcare industry [4,18,19]. It has also explained regulatory policy trends related to digital healthcare [15,20,21]. In addition, several studies have focused on the technical aspects of digital healthcare [9,10,11,12,13].

### 2.2. Data-Driven Healthcare-Related Studies

Data-oriented digitization is particularly expected to accelerate in the healthcare sector. Studies related to healthcare data continue to be published, and previous studies in South Korea have fallen mainly into three categories. First, there are studies related to healthcare data collection or storage [22,23]. Nowadays huge amounts of healthcare data are being generated through various channels, so the optimal way in which to collect, store, and manage large amounts of data is an important issue [24,25], and innovative tools and powerful technologies are required to address it [26]. In this context, some studies in South Korea have focused on technical aspects such as collecting, integrating, and storing information, with the goal of improving healthcare services [22,23,27].

Second, there have been several studies on the laws and policies related to data security and privacy [20,28,29]. These studies have discussed how to regulate and monitor sensitive personal health information, with the aim of preventing the infringement of people’s privacy [28]. Some researchers have investigated the issues surrounding the recent revisions of three South Korean laws related to the protection of personal information, namely the Personal Information Protection Act, the Credit Information Act, and the Information and Communications Network Act [28,29]. Although the revision of the Personal Information Protection Act may promote data utilization, it might be of limited use in encouraging the revitalization of the data-driven economy [29].

Third, in addition to research on laws and regulations related to healthcare data, studies on technology development aimed at enhancing data security and protecting privacy have been conducted. For example, a method of collecting and analyzing personal health information through de-identification of healthcare data has been developed [30]. In addition, a new model for the safe processing of medical information on mobile devices has been proposed [31].

Today, many experts agree that data are the driver of digital healthcare innovation, on the basis of multiple strands of research on digital healthcare which have adopted a variety of approaches across a range of academic fields. The sheer volume of healthcare data is expected to continue to increase, and various new services could be provided based on these data [7,19]. In other words, data will play a key role in a range of fields in the healthcare sector. However, although some scholars in various academic fields have investigated issues related to healthcare data, there still remain significant gaps in the research. As discussed above, most studies have focused only on a specific area, such as the legal or policy framework of healthcare data, or the technical issues involved. However, the potential future of the healthcare data-driven economy varies significantly depending on how the various factors surrounding healthcare data are connected and interact with each other. Nevertheless, few studies have systematically analyzed and taken stock of these various potential future developments in the healthcare data-driven economy. In addition, many studies related to digital healthcare in the field of social science in South Korea remain merely descriptive and exploratory. To overcome the limitations of this existing research, this paper analyzes how Korea’s data-driven healthcare economy could unfold in the future, using the scenario technique.

## 3. Methods

### 3.1. Scenario Modelling through General Morphological Analysis (GMA)

The scenario technique can be particularly useful in predicting the future of the data-based healthcare economy. To deal with the uncertain future and prepare for unexpected situations, research on the potential future can provide the knowledge needed in the present and evaluate the plausibility of future outcomes [32]. The scenario technique is a systematic way of presenting combinations of situations that may occur in the future. It shows how future situations can come about in many different ways, so it can create a suitable strategy for addressing them. Consequently, the scenario technique can be said to perform a bridging operation which links predictions about the future with preparatory strategies.

As a method employed in the scenario technique, GMA has received attention along with trend analysis, cross-impact analysis, and Battelle Scenario Inputs to Corporate Strategies (BASICS). GMA was developed by Swiss astrophysicist and aerospace scientist Fritz Zwicky. He attempted to identify the comprehensive relationships involved in complex phenomena or ideas, and developed a more simplified interrelation construction. GMA is a method that structures the multidimensional relations between objects. It deconstructs systems into sub-systems, and systematically classifies combinations of sub-systems. Quantitative methods and the identification of causal relations have difficulty in dealing with human behavior and political decisions that necessitate normative judgement and cause unpredictable outcomes. However, GMA can examine these dimensions by breaking them down into sub-systems. In this context, Zwicky considers this method as a form of ‘totality research’ [33].

It has been applied to various areas of astrophysics such as the development of rocket propulsion, object classification, the legal issues surrounding space travel, and space colonization. It has also contributed to the academic fields of linguistics, zoology, botany, mathematics, engineering, architecture, and defense research. In particular, it is utilized in policy analysis and research on the future. For example, using GMA, Coyle and McGlone developed several future scenarios with projected problems that South-east Asia and the South-west Pacific could face [34]. Since then, morphological analysis has been employed as an analytical tool in relation to the structuring of complicated areas of policy in many countries such as the United States, South Africa, and Australia, as well as in several European countries [35,36,37,38].

Healthcare studies using GMA in South Korea are comparatively rare, although it has been employed as a method of developing a new u-healthcare service model [8]. Based on a literature review, Hwang and her colleagues defined the dimensions and elements that form the u-healthcare service and created a morphological box. They have expanded the morphological space by adding new elements and analyzed the new u-healthcare services available through a combination between them. As a result, this study suggested four new u-healthcare services—namely patient medical information service, u-home medical service, home care service, and personalized monitoring service—might be developed in the u-IT environments.

### 3.2. Multi-Dimensional Scaling (MDS)

GMA requires the resetting of complex multi-dimensional data, which is accomplished by reducing them to several simple dimensions. At the same time, it must include information derived from the original data to the greatest extent possible. Therefore, in order to reduce the quantity of data involved, various scaling or reduction tools can be applied to GMA, including principal component analysis, factor analysis, multi-dimensional scaling, cluster analysis, and semantic mapping. In this study, we applied multidimensional scaling (MDS) to GMA. This is a generalized method that identifies concealed structures by simplifying complex variable information. This operation categorizes variables into only a few dimensions and demonstrates the relationships between them. In this way, researchers can demonstrate latent similarity and dissimilarity among entities [39]; that is, the method is used to discover meaningful underlying dimensions and to reveal latent structures by simplifying variable information.

## 4. Results

### 4.1. Expert Evaluation and Results of GMA

First, 11 analysts were selected from a pool of experts from the Korea Institute of Science and Technology Evaluation and Planning (KISTEP). We strove to choose these experts from as wide a variety of healthcare data fields as possible in order to define the problems that would be structured with GMA. The selected expert opinion group consisted of professors, doctors, and researchers in the fields of the information society, future research, science and technology policies, medical and healthcare issues, and healthcare policies. In the first workshop, the experts shared their opinions on the impact of research thus far, the comprehensive context of the data-driven healthcare economy, and the composition of the optimal research model. Additionally, in the second workshop, major factors (variables) in the data-driven healthcare economy were derived, and a matrix was constructed. Finally, the experts performed a cross-consistency assessment of the matrix.

**Figure 1:**
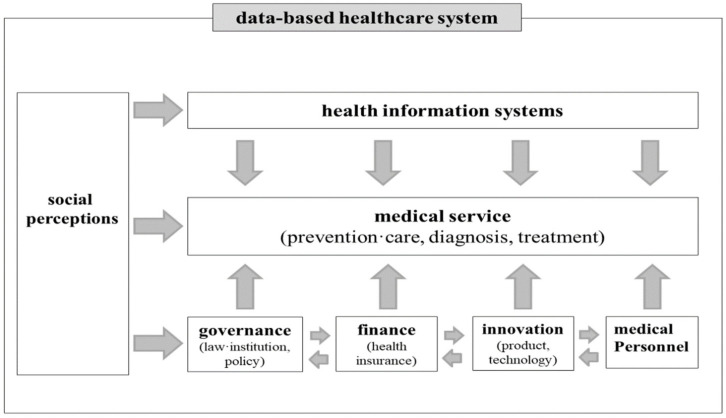
Data-based healthcare system.

Figure 1 shows the data-based healthcare system that was devised in the course of a series of expert workshops. This system was developed on the basis of the various components of the WHO health systems framework [40]. Additionally, key factors involved in the data-driven healthcare economy scenarios of the future were derived based on the components of this system, as shown in Figure 1. In other words, the core factors involved in the various scenarios consist of the detailed issues related to each component in Figure 1. In order to develop future scenarios of the healthcare data-driven economy, the following seven factors were selected: the acceptability of data utilization, data literacy, healthcare data regulation, healthcare data system, medical costs, the convergence of ICT and biotechnology, and the utilization of data in medical services. The acceptability of data utilization and the level of data literacy are factors that correspond to social perceptions. Additionally, the healthcare data system, the utilization of data in medical services, healthcare data regulation, medical costs, and the convergence of ICT and biotechnology are pertinent to the issues of the health information system, medical services, governance, financing, and innovation in Figure 1, respectively.

The acceptability of data utilization indicates the extent to which society or its members accept the use of data containing personal information. Data literacy refers to how widespread the ability to use data to solve problems is, based on people’s overall knowledge and understanding of data, such as its context and structure [41]. In other words, it means the ability to effectively understand and use data in decision making [42]. Healthcare data regulation refers to the reach and effectiveness of national data protection regulations. A healthcare data system is one which is built for the production, collection, storage, and utilization of healthcare data. The increase or decrease in medical expenses that may occur in the future is also an important factor in this area. Additionally, the convergence of ICT and biotechnology is necessary to ensure the provision of effective medical services such as optimized health checkups and disease prevention interventions to individuals. The final factor, the utilization of data in medical services, denotes the employment of data in real-world medical services.

Table 1 shows the spectrum of degrees of possible change that may be undergone by each factor in the future. Too many spectral values corresponding to key factors can complicate the composition of a scenario. Therefore, in this study, the number of spectral values was limited to simplify the various scenarios. Except for the convergence of ICT and biotechnology, the contents of the spectrum of the six other factors represent an ordinal variable rather than a nominal one. Each of these six factors was seen as having only three potential futures: strengthening, weakening, or stasis.

To conduct a morphological analysis, a cross-consistency assessment was first performed by the expert group in order to evaluate whether the internal relationships between the concepts involved were contradictory or logically consistent. In other words, the association between two spectral values was evaluated. Associations that were considered to be logically or empirically inconsistent were coded as 0, modest associations as 1, and strong associations as 2. Additionally, self-association was coded as 4 instead of 3 in order to develop a relatively distinct scenario. The mean value of the resulting cross-consistency assessment is shown in Table 2. A matrix that indicates the distance between spectral values, obtained by processing the values in Table 2 in order to perform the morphological analysis, has been included in the Appendix A. A cross-consistency assessment is regarded as a means of identifying kinds of association; for this purpose, a matrix composed of values obtained by dividing the other associations by the self-association value of 4 was used. In addition, Table 3 shows the mode of cross-consistency assessment conducted by the experts, which produced the most frequently identified value. The matrix of the distances between spectral values is also included in the Appendix B. Although the specific distances vary somewhat, the distances between mean spectral values and those between the modes of spectral values display a substantially similar pattern.

### 4.2. Results of MDS

**Figure 2:**
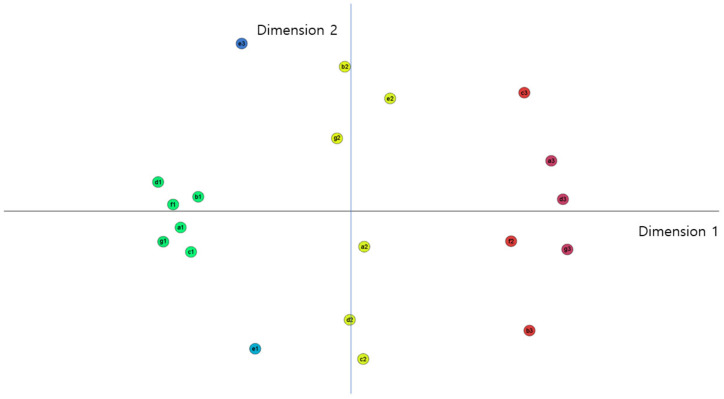
Results of MDS (expert evaluation mean). 1: Standardized circle stress: 0.067; explained dispersion: 0.932; Tucker’s coefficient of congruence: 0.966; 2: a—acceptability of data utilization; b—data literacy; c—healthcare data regulation; d—healthcare data system; e—medical costs; f—convergence of ICT and biotechnology; g—utilization of data in medical services.

After conducting a GMA on the basis of the matrix consisting of expert evaluation mean values, this study used multidimensional scaling for future scenario deduction. Through MDS, the relations among the spectra of sub-systems in the data-driven healthcare economy were demonstrated in two-dimensional plots. This study derived several scenarios by distinguishing structural patterns in simplified and visualized data. The result of the MDS plot is displayed in Figure 2. The coordinates of the spectra are shown in Table 4. The statistics for goodness of fit in multidimensional scaling are as follows: standardized circle stress: 0.067; explained dispersion: 0.932; and Tucker’s coefficient of congruence: 0.966. This implies that model fitness is 0.932, and 93% of the variation can be explained in cases where two dimensions are involved.

Four scenarios can be constructed based on coordinate and visualization information (Table 5). We named the first the optimistic scenario. It indicates optimistic spectra and the high association among them. Specifically, it includes the expanded acceptability of data utilization, a high level of data literacy, weakened healthcare data regulation, a fully realized healthcare data system, increased medical costs, the active convergence of ICT and biotechnology, and the expanded utilization of data in medical services. The second scenario is mostly similar to the first one, and includes the expanded acceptability of data utilization, a high level of data literacy, weakened healthcare data regulation, a fully realized healthcare data system, the active convergence of ICT and biotechnology, and expanded utilization of data in medical services. However, unlike the first scenario, medical costs may decrease in the second scenario; that is, the second scenario could be a more desirable one. Overall, the third scenario, with relatively pessimistic spectra, contrasts with the first. It includes the contraction of data utilization, a low level of data literacy, reinforced healthcare data regulation, a deactivated healthcare data system, unchanged medical costs, the stagnation of ICT and biotechnology, and the reduced utilization of data in medical services. Therefore, we call this the pessimistic scenario. The final scenario displays intermediate spectrum and has the lowest level of association among the spectra, so we call it the monotonous scenario.

**Figure 3:**
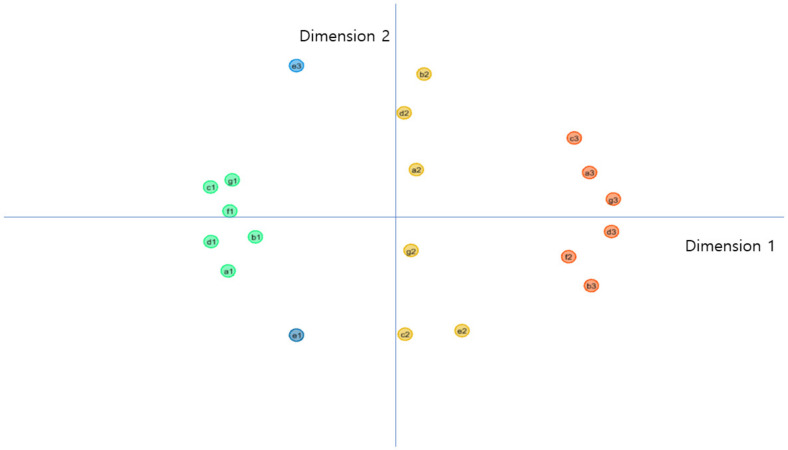
Results of MDS (expert evaluation mode). 1: Standardized circle stress: 0.0567; explained dispersion: 0.944; Tucker’s coefficient of congruence: 0.971; 2: a—acceptability of data utilization; b—data literacy; c—healthcare data regulation; d—healthcare data system; e—medical costs; f—convergence of ICT and biotechnology; g—utilization of data in medical services.

In the case of the expert evaluation mode, the result of the MDS plot is displayed in Figure 3. Each coordinate in two dimensions is illustrated in Table 6. The statistics for goodness of fit are as follows: standardized circle stress; 0.0567; explained dispersion: 0.944; and Tucker’s coefficient of congruence: 0.971. This means that model fitness is 0.971, and 97% of the variation can be explained in two dimensions. The result of the GMA derived from the mode of expert evaluation (Table 7) is similar to that derived from the mean of expert evaluation. 

In summary, the first scenario envisions mostly optimistic prospects, entailing close associations among spectra, and the second scenario is mostly similar to the first, but differs in that it envisions medical costs being reduced. The third scenario displays pessimism, and the contents of the final scenario indicate the maintenance of the status quo. Therefore, based on expert evaluations, these results show that the future healthcare data economy can take various forms.

## 5. Conclusions

With the development of digital technology, the healthcare industry has emerged as a new growth engine that creates new value, as it has expanded from discovering cures for diseases to the areas of prevention and care. In particular, the significance of data-based healthcare systems in easing the healthcare burden on national finances and improving the quality of healthcare in South Korea, where national medical expenses are rapidly rising due to the aging population and the increase in chronic diseases, is ever clearer. Although researchers, healthcare officials, and policymakers have shown interest in data-based healthcare innovation, which can provide optimized healthcare services to individuals by recording, integrating, analyzing, and utilizing healthcare data in Korea, little attention has been paid to forecasting the parameters of the future data-driven healthcare economy. This study analyzed possible future scenarios of this economy in South Korea through general morphological analysis.

Seven major factors which will constitute the future of the data-driven healthcare economy were selected, and a spectrum indicating the degree of change of each dimension was determined through expert workshops. Through multidimensional scaling, four scenarios were derived for the data-driven healthcare economy of the future, according to the prospects of the healthcare system and the levels of association between the spectra of each factor. The first scenario suggests mostly optimistic prospects, as it envisions the expanded acceptability of data utilization, a high rate of data literacy, weakened healthcare data regulation, a fully realized healthcare data system, the convergence of ICT and biotechnology, and expanded utilization of data in medical services, although it also entails increased medical costs. The second scenario is mostly similar to the first one except for the reduction in medical costs it involves. Meanwhile, the third scenario foresees relatively pessimistic prospects, as in this scenario, there is a low level of acceptability of data utilization and of data literacy, healthcare data regulation is reinforced, the healthcare data system is deactivated, medical costs are maintained at existing levels, the convergence of ICT and biotechnology slows, and the utilization of data in medical services diminishes. In the final scenario, most factors remain unchanged, persisting at their current levels. In summary, various scenarios were created according to the degree of change in the seven core factors.

This study has several policy implications on the basis of an analysis of possible situations that may come about in the future in relation to healthcare data. If Scenario 1 or Scenario 2 arises in the future, it may be necessary to expand government-level support for companies in the healthcare sector in relation to the use of data and through new healthcare data development projects. Additionally, it may be necessary to consider the needs of vulnerable people in terms of their capacity to utilize their medical data. However, in the case of Scenario 1, policy efforts will be required to expand public health insurance and to reduce the medical expenses associated with various digital healthcare services. In Scenario 3 and Scenario 4, government efforts to expand R&D support for digital healthcare, including ICT and BIO convergence, will be needed. Additionally, it will be necessary to improve people’s data literacy and to train professionals in various fields related to digital healthcare. In addition, in Scenario 4, efforts to secure digital healthcare data at the national level, and to review and improve the legal system in order to optimize the use of information in the medical field, may be necessary.

In particular, threats to privacy may become an important issue in situations where healthcare data regulations are eased, healthcare data systems are realized, and data use is expanded in medical services, such as in Scenario 1 and Scenario 2. The risks posed to patients by incorrect treatment decisions based on their data, and the abuse and intentional manipulation of this data, are real. There is a need for the development of big data technologies that can utilize and employ medical data under strict privacy guarantees. Data-driven healthcare technologies can be dangerous if patients cannot ensure their own privacy [43]. Overall, in a data-driven economy, who controls the data through activities such as data collection and use, is a crucial issue [44]. The role of the government will be very important in balancing the economic and other interests of all stakeholders in the healthcare arena.

This study also has academic contributions to make. First, its use of the concept of the data-driven healthcare economy is important. Second, most of the existing healthcare studies focus on current policies and technological issues. However, our paper is significant in that it focuses on the future data-driven healthcare economy. Additionally, it employs the scenario technique, a future-oriented research method, in order to predict this future. Finally, this study, which analyzes the situation in South Korea, can also contribute to the field of healthcare research more widely in East Asia.

Despite these contributions, our study has some limitations. Although we examine the future data-driven healthcare economy on the basis of four distinct scenarios, which in turn are based on seven core healthcare dimensions, an even more complex picture could still emerge in future research. In addition, by imagining novel combinations of spectrum values, other possible futures of the healthcare data economy could be identified. Additionally, since this paper outlines possible futures of the data-driven healthcare economy in South Korea, it cannot be applied wholesale to other countries, as possible scenarios may vary from country to country.

## Figures and Tables

**Table 1:** Key factors in the future of data-based healthcare economy and spectrum.

Key Factors	Acceptability of Data Utilization	Data Literacy	Healthcare Data Regulation	Healthcare Data System	Medical Costs	Convergence of ICT and Biotechnology	Utilization of Data in Medical Services
Spectrum	Expansion	High	Weakening	Realization	Increase	Realization	Expansion
	Status Quo	Status Quo	Status Quo	Status Quo	Status Quo		Status Quo
	Contraction	Low	Reinforcement	Deactivation	Decrease	Stagnation	Reduction

**Table 2:** Expert evaluation (mean).

Expert Evaluation (Mean)		Acceptability of Data Utilization			Data Literacy			Healthcare Data Regulation			Healthcare Data System			Medical Costs			Convergence of ICT and Biotechnology		Utilization of Data in Medical Services		
		Expansion	Status Quo	Contraction	High	Status Quo	Low	Weakening	Status Quo	Reinforcement	Realization	Status Quo	Deactivation	Increase	Status Quo	Decrease	Realization	Stagnation	Expansion	Status Quo	Reduction
Acceptability of Data Utilization	Expansion																				
	Status Quo																				
	Contraction																				
Data Literacy	High	2.0	1.1	0.7																	
	Status Quo	1.3	1.1	0.8																	
	Low	0.5	0.6	1.5																	
Healthcare Data Regulation	Weakening	2.0	1.1	0.6	1.7	0.9	0.6														
	Status Quo	1.2	1.1	0.9	0.9	1.2	0.7														
	Reinforcement	0.2	0.7	1.7	1.1	0.7	0.9														
Healthcare Data System	Realization	2.0	0.9	0.2	2.0	1.0	0.2	1.8	1.1	0.5											
	Status Quo	1.0	1.5	0.6	0.9	1.5	0.7	1.1	1.5	0.5											
	Deactivation	0.2	0.8	1.9	0.2	0.8	1.9	0.5	0.8	1.6											
Medical Costs	Increase	1.5	0.9	0.5	1.2	1.0	0.9	1.4	0.9	0.7	1.4	1.0	0.5								
	Status Quo	1.2	1.1	0.6	1.0	1.2	0.5	0.9	1.1	0.7	1.0	1.1	0.6								
	Decrease	1.2	1.0	0.6	0.9	1.0	0.5	0.8	0.9	0.5	1.0	0.9	0.5								
Convergence of ICT and Biotechnology	Realization	1.8	0.8	0.5	2.0	1.0	0.2	1.9	1.1	0.4	2.0	0.9	0.2	1.5	1.0	1.0					
	Stagnation	0.5	1.0	1.4	0.5	0.9	1.5	0.5	1.0	1.4	0.2	0.9	1.7	0.9	1.2	0.5					
Utilization of Data in Medical Services	Expansion	2.0	1.0	0.3	2.0	0.9	0.5	2.0	1.0	0.3	2.0	0.9	0.1	1.5	1.0	1.4	2.0	0.3			
	Status Quo	1.1	1.3	0.5	1.1	1.2	0.6	1.1	1.1	0.8	1.0	1.3	1.0	1.0	1.2	1.0	0.9	0.9			
	Reduction	0.1	0.8	1.7	0.4	0.9	1.5	0.2	0.7	1.4	0.0	0.7	1.6	0.9	0.7	0.7	0.1	1.6			

**Table 3:** Expert evaluation (mode).

Expert Evaluation (Mode)		Acceptability of Data Utilization			Data Literacy			Healthcare Data Regulation			Healthcare Data System			Medical Costs			Convergence of ICT and Biotechnology		Utilization of Data in Medical Services		
		Expansion	Status Quo	Contraction	High	Status Quo	Low	Weakening	Status Quo	Reinforcement	Realization	Status Quo	Deactivation	Increase	Status Quo	Decrease	Realization	Stagnation	Expansion	Status Quo	Reduction
Acceptability of Data Utilization	Expansion																				
	Status Quo																				
	Contraction																				
Data Literacy	High	2	1	0																	
	Status Quo	1	1	1																	
	Low	0	1	2																	
Healthcare Data Regulation	Weakening	2	1	0	2	1	0														
	Status Quo	1	1	1	1	1	1														
	Reinforcement	0	1	2	1	1	1														
Healthcare Data System	Realization	2	1	0	2	1	0	2	1	0											
	Status Quo	1	1	1	1	1	1	1	1	1											
	Deactivation	0	1	2	0	1	2	0	1	2											
Medical Costs	Increase	2	1	1	1	1	1	1	1	0	1	1	0								
	Status Quo	1	1	1	1	1	1	1	1	1	1	1	1								
	Decrease	1	1	1	1	1	0	1	1	1	1	1	0								
Convergence of ICT and Biotechnology	Realization	2	1	0	2	1	0	2	1	0	2	1	0	1	1	1					
	Stagnation	0	1	1	1	1	2	0	1	2	0	1	2	1	1	0					
Utilization of Data in Medical Services	Expansion	2	1	0	2	1	0	2	1	0	2	1	0	2	1	2	2	0			
	Status Quo	1	1	1	1	1	1	1	1	1	1	1	1	1	1	1	1	1			
	Reduction	0	1	2	0	1	2	0	1	2	0	1	2	0	1	0	0	2			

**Table 4:** Coordinates of spectra (expert evaluation mean).

Coordinates of Spectra (Expert Evaluation Mean)			Dimension	
			1	2
Acceptability of Data Utilization	Expansion	a1	−0.645	−0.070
	Status Quo	a2	0.063	−0.147
	Contraction	a3	0.785	0.197
Data Literacy	High	b1	−0.577	0.053
	Status Quo	b2	−0.012	0.574
	Low	b3	0.701	−0.483
Healthcare Data Regulation	Weakening	c1	−0.605	−0.168
	Status Quo	c2	0.059	−0.597
	Reinforcement	c3	0.681	0.470
Healthcare Data System	Realization	d1	−0.732	0.112
	Status Quo	d2	0.007	−0.440
	Deactivation	d3	0.830	0.043
Medical Costs	Increase	e1	−0.358	−0.556
	Status Quo	e2	0.163	0.447
	Decrease	e3	−0.410	0.667
Convergence of ICT and Biotechnology	Realization	f1	−0.674	0.022
	Stagnation	f2	0.630	−0.125
Utilization of Data in Medical Services	Expansion	g1	−0.712	−0.127
	Status Quo	g2	−0.042	0.287
	Reduction	g3	0.847	−0.158

**Table 5:** Result of GMA scenario (expert evaluation mean).

Key Factors	Acceptability of Data Utilization	Data Literacy	Healthcare Data Regulation	Healthcare Data System	Medical Costs	Convergence of ICT and Biotechnology	Utilization of Data in Medical Services
Spectrum	expansion	high	Weakening	Realization	increase	Realization	expansion
					
	Status Quo	Status Quo	Status Quo	Status Quo	Status Quo	stagnation	Status Quo
	
	Contraction	low	reinforcement	Deactivation	decrease		reduction

Note: 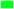
—Scenario 1: Optimistic Scenario; 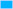
—Scenario 2: Desirable Scenario; 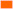
—Scenario 3: Pessimistic Scenario; 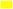
—Scenario 4: Monotonous Scenario.

**Table 6:** Coordinates of spectra (expert evaluation mode).

Coordinates of Spectra (Expert Evaluation Mode)			Dimension	
			1	2
Acceptability of Data Utilization	Expansion	a1	−0.680	−0.255
	Status Quo	a2	0.058	0.200
	Contraction	a3	0.737	0.187
Data Literacy	High	b1	−0.573	−0.102
	Status Quo	b2	0.088	0.629
	Low	b3	0.744	−0.321
Healthcare Data Regulation	Weakening	c1	−0.749	0.122
	Status Quo	c2	0.014	−0.539
	Reinforcement	c3	0.677	0.342
Healthcare Data System	Realization	d1	−0.747	0.123
	Status Quo	d2	0.010	0.455
	Deactivation	d3	0.823	−0.078
Medical Costs	Increase	e1	−0.412	−0.543
	Status Quo	e2	0.237	−0.523
	Decrease	e3	−0.412	0.667
Convergence of ICT and Biotechnology	Realization	f1	−0.673	0.014
	Stagnation	f2	0.655	−0.191
Utilization of Data in Medical Services	Expansion	g1	−0.664	0.154
	Status Quo	g2	0.037	−0.163
	Reduction	g3	0.830	0.068

**Table 7:** Results of GMA scenario (expert evaluation mode).

Key Factors	Acceptability of Data Utilization	Data Literacy	Healthcare Data Regulation	Healthcare Data System	Medical Costs	Convergence of ICT and Biotechnology	Utilization of Data in Medical Services
Spectrum	expansion	High	Weakening	Realization	increase	Realization	expansion
					
	Status Quo	Status Quo	Status Quo	Status Quo	Status Quo	stagnation	Status Quo
	
	Contraction	low	reinforcement	Deactivation	decrease		reduction

Note: 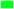
—Scenario 1: Optimistic Scenario; 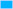
—Scenario 2: Desirable Scenario; 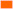
—Scenario 3: Pessimistic Scenario; 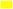
—Scenario 4: Monotonous Scenario.

## Data Availability

The data presented in this study are available upon request from the corresponding author.

## Appendix A

**Table A1:** Distance between Spectra (Expert Evaluation Mean).

Distance between Spectra (Expert Evaluation Mean)		Acceptability of Data Utilization			Data Literacy			Healthcare Data Regulation			Healthcare Data System			Medical Costs			Convergence of ICT and Biotechnology		Utilization of Data in Medical Services		
		Expansion	Status Quo	Contraction	High	Status Quo	Low	Weakening	Status Quo	Reinforcement	Realization	Status Quo	Deactivation	Increase	Status Quo	Decrease	Realization	Stagnation	Expansion	Status Quo	Reduction
Acceptability of Data Utilization	Expansion	0.000																			
	Status Quo	0.712	0.000																		
	Contraction	1.454	0.799	0.000																	
Data Literacy	High	0.141	0.671	1.369	0.000																
	Status Quo	0.903	0.725	0.882	0.768	0.000															
	Low	1.408	0.721	0.686	1.386	1.275	0.000														
Healthcare Data Regulation	Weakening	0.106	0.669	1.437	0.224	0.950	1.343	0.000													
	Status Quo	0.880	0.450	1.076	0.910	1.173	0.652	0.791	0.000												
	Reinforcement	1.431	0.873	0.293	1.325	0.700	0.954	1.436	1.235	0.000											
Healthcare Data System	Realization	0.201	0.837	1.520	0.166	0.856	1.552	0.308	1.062	1.458	0.000										
	Status Quo	0.749	0.298	1.006	0.764	1.014	0.696	0.669	0.166	1.133	0.922	0.000									
	Deactivation	1.479	0.790	0.161	1.407	0.996	0.542	1.451	1.002	0.453	1.564	0.955	0.000								
Medical Costs	Increase	0.564	0.588	1.369	0.648	1.182	1.062	0.460	0.420	1.461	0.765	0.383	1.331	0.000							
	Status Quo	0.959	0.602	0.670	0.838	0.216	1.075	0.984	1.049	0.518	0.956	0.900	0.781	1.131	0.000						
	Decrease	0.774	0.942	1.284	0.636	0.408	1.599	0.858	1.349	1.108	0.643	1.183	1.389	1.225	0.613	0.000					
Convergence of ICT and Biotechnology	Realization	0.096	0.756	1.469	0.102	0.861	1.465	0.203	0.960	1.426	0.107	0.822	1.504	0.659	0.938	0.697	0.000				
	Stagnation	1.276	0.567	0.358	1.220	0.949	0.365	1.235	0.740	0.598	1.382	0.698	0.262	1.078	0.739	1.307	1.312	0.000			
Utilization of Data in Medical Services	Expansion	0.088	0.775	1.531	0.225	0.990	1.457	0.115	0.903	1.515	0.239	0.784	1.551	0.556	1.046	0.849	0.154	1.341	0.000		
	Status Quo	0.700	0.447	0.832	0.583	0.289	1.070	0.724	0.890	0.746	0.712	0.729	0.906	0.901	0.260	0.529	0.685	0.788	0.787	0.000	
	Reduction	1.494	0.783	0.361	1.439	1.129	0.356	1.452	0.902	0.650	1.602	0.886	0.202	1.269	0.913	1.503	1.531	0.219	1.559	0.994	0.000

## Appendix B

**Table A2:** Distance between Spectra (Expert Evaluation Mode).

Distance between Spectra (Expert Evaluation Mode)		Acceptability of Data Utilization			Data Literacy			Healthcare Data Regulation			Healthcare Data System			Medical Costs			Convergence of ICT and Biotechnology		Utilization of Data in Medical Services		
		Expansion	Status Quo	Contraction	High	Status Quo	Low	Weakening	Status Quo	Reinforcement	Realization	Status Quo	Deactivation	Increase	Status Quo	Decrease	Realization	Stagnation	Expansion	Status Quo	Reduction
Acceptability of Data Utilization	Expansion	0.000																			
	Status Quo	0.867	0.000																		
	Contraction	1.484	0.678	0.000																	
Data Literacy	High	0.187	0.699	1.341	0.000																
	Status Quo	1.171	0.430	0.785	0.985	0.000															
	Low	1.426	0.861	0.508	1.335	1.155	0.000														
Healthcare Data Regulation	Weakening	0.383	0.811	1.487	0.285	0.979	1.558	0.000													
	Status Quo	0.749	0.740	1.024	0.732	1.170	0.762	1.009	0.000												
	Reinforcement	1.482	0.635	0.166	1.326	0.655	0.667	1.443	1.103	0.000											
Healthcare Data System	Realization	0.148	0.867	1.515	0.175	1.123	1.504	0.244	0.867	1.497	0.000										
	Status Quo	0.990	0.259	0.774	0.806	0.191	1.068	0.829	0.994	0.676	0.952	0.000									
	Deactivation	1.513	0.814	0.279	1.396	1.020	0.256	1.585	0.931	0.445	1.571	0.972	0.000								
Medical Costs	Increase	0.394	0.879	1.361	0.470	1.275	1.177	0.746	0.425	1.403	0.538	1.084	1.320	0.000							
	Status Quo	0.955	0.744	0.868	0.913	1.162	0.546	1.178	0.224	0.970	1.062	1.004	0.736	0.649	0.000						
	Decrease	0.960	0.664	1.246	0.786	0.502	1.522	0.641	1.279	1.137	0.858	0.473	1.443	1.211	1.356	0.000					
Convergence of ICT and Biotechnology	Realization	0.269	0.754	1.420	0.153	0.978	1.456	0.132	0.882	1.389	0.156	0.813	1.499	0.616	1.057	0.703	0.000				
	Stagnation	1.336	0.713	0.387	1.231	0.997	0.158	1.438	0.729	0.534	1.403	0.912	0.203	1.123	0.534	1.370	1.343	0.000			
Utilization of Data in Medical Services	Expansion	0.409	0.724	1.401	0.272	0.890	1.487	0.091	0.969	1.354	0.289	0.739	1.505	0.742	1.127	0.572	0.140	1.363	0.000		
	Status Quo	0.722	0.364	0.783	0.613	0.794	0.725	0.836	0.376	0.815	0.785	0.618	0.791	0.588	0.412	0.944	0.731	0.619	0.769	0.000	
	Reduction	1.543	0.782	0.151	1.413	0.930	0.398	1.580	1.017	0.314	1.588	0.906	0.146	1.384	0.837	1.379	1.503	0.313	1.496	0.826	0.000
